# OPTIMISING THE PARAMETERS OF COCHLEAR IMPLANT IMAGING WITH CONE-BEAM COMPUTED TOMOGRAPHY

**DOI:** 10.1093/rpd/ncad019

**Published:** 2023-02-13

**Authors:** Samuel Söderqvist, Ville Sivonen, Antti Aarnisalo, Harri Karppi, Saku T Sinkkonen, Juha Koivisto

**Affiliations:** Department of Otorhinolaryngology—Head and Neck Surgery, Head and Neck Center, Helsinki University Hospital and University of Helsinki, Helsinki, Finland; Tauno Palva Laboratory, Department of Otorhinolaryngology—Head and Neck Surgery, Head and Neck Center, Helsinki University Hospital, Helsinki, Finland; Department of Otorhinolaryngology—Head and Neck Surgery, Head and Neck Center, Helsinki University Hospital and University of Helsinki, Helsinki, Finland; Department of Otorhinolaryngology—Head and Neck Surgery, Head and Neck Center, Helsinki University Hospital and University of Helsinki, Helsinki, Finland; Planmeca Oy, Helsinki, Finland; Department of Otorhinolaryngology—Head and Neck Surgery, Head and Neck Center, Helsinki University Hospital and University of Helsinki, Helsinki, Finland; Tauno Palva Laboratory, Department of Otorhinolaryngology—Head and Neck Surgery, Head and Neck Center, Helsinki University Hospital, Helsinki, Finland; Department of Physics, University of Helsinki, Helsinki, Finland

## Abstract

With computed tomography (CT), the delicate structures of the inner ear may be hard to visualise, which a cochlear implant (CI) electrode array can further complicate. The usefulness of a novel cone-beam CT device in CI recipient’s inner ear imaging was evaluated and the exposure parameters were optimised to attain adequate clinical image quality at the lowest effective dose (ED). Six temporal bones were implanted with a Cochlear Slim Straight electrode array and imaged with six different imaging protocols. Contrast-to-noise ratio was calculated for each imaging protocol, and three observers evaluated independently the image quality of each imaging protocol and temporal bone. The overall image quality of the inner ear structures did not differ between the imaging protocols and the most relevant inner ear structures of CI recipient’s inner ear can be visualised with a low ED. To visualise the most delicate structures in the inner ear, imaging protocols with higher radiation exposure may be required.

## Introduction

The cochlear implant (CI) has become the standard hearing-rehabilitation method for patients suffering from severe-to-profound hearing loss. Before the CI surgery, preoperative computed tomography (CT) and magnetic resonance imaging are routinely performed to assess cochlear and temporal bone anatomy. Also, there are several CI-related issues requiring intraoperative and postoperative imaging, which are often performed by CT. The correct placement of the CI electrode array in the scala tympani (ST) is crucial for appropriate benefit from the device. However, abnormal anatomy or electrode array tip fold-over, scalar translocation from the ST to the scala vestibuli (SV) or incomplete electrode array insertion might compromise patient outcomes.

Currently, the gold standard for the inner ear imaging is multidetector CT (MDCT). However, the cone-beam CT (CBCT) technology might provide several benefits over MDCT. While the CBCT image quality of the inner ear imaging is at least similar to MDCT imaging^(1, 2)^, CBCT scans cause lower effective doses (ED) when compared with scans with MDCT^(2–5)^.

The ED of CBCT imaging varies depending on the scanner, the field of view (FOV) size, and the imaging parameters. Choosing the imaging parameters is always a compromise between the radiation exposure and the image quality. By optimising the tube voltage (kVp) and current exposure time product (mAs), sufficient image quality for radiological diagnostics can be achieved with minimal radiation exposure to the patient. Even though the temporal bone region is ideal for low-dose CT imaging^(6)^, the image quality might not be sufficient for evaluation of the most delicate structures, such as the stapes or the modiolus of the cochlea^(1, 6)^. Therefore, parameter optimisation is especially crucial for the imaging of the inner ear, as low radiation intensity increases the noise in the small voxels required to visualise the delicate structures^(7)^. The noise can be reduced, together with an increase of the ED, by increasing the mAs, the kVp or both. While the ED increases linearly with mAs, the relationship between the ED and the kVp is more intricate, as the complex interactions between the photons and the tissue are affected by the energy of the X-ray beam^(8)^.

The intracochlear part of a CI is a multielectrode array, which consists of platinum electrodes causing metal artifacts in the CBCT images, potentially debilitating their image quality. The metal artifact created by the platinum electrodes accounts for 50–70% of the electrode diameter measured from the CBCT images, and is consistent between different radiation doses^(9)^. However, the clinical usefulness of CBCT in the imaging of CI recipient’s inner ear has been demonstrated both in vitro^(10)^ and *in vivo*^(11)^. As low as diagnostically acceptable principle recommends to expose the patient to the minimum amount of radiation required for sufficient image quality for a diagnosis^(12)^. If the image quality is not adequate for a diagnosis, the patient is exposed to radiation in vain. On the other hand, every additional ED caused by attaining images too precise are as unwanted. Thus, the purpose of this study was to optimise the novel full body CBCT device exposure parameters for imaging of the inner ear implanted with a CI and to attain adequate clinical image quality at the lowest ED.

## Materials and methods

### Study design and ethics

This prospective cadaver temporal bone study was approved by the institutional review board of the Hospital District of Helsinki and Uusimaa. The six anonymous cadaver temporal bones used in this study were dissected with the permission of Valvira in the Finnish Institute for Health and Welfare’s Department of Forensic Medicine.

### Preparation of the temporal bones

A mastoidectomy and posterior tympanotomy was performed on each of the six temporal bones with a similar approach as in clinical practice with CI recipients at the Tauno Palva Temporal Bone Laboratory in the Department of Otorhinolaryngology—Head and Neck Surgery, Head and Neck Center, Helsinki University Hospital and transferred to Planmeca Oy for further procedures. To fill the cochleae with fluid similar to the perilymph, the temporal bones were submerged in Ringer’s solution bath and subsequently placed in a vacuum for a complete fill and removal of any air bubbles inside the cochlea. After confirming the absence of air bubbles via CBCT imaging, each cochlea was implanted with a Cochlear Slim Straight (Cochlear Ltd, Sydney, Australia) electrode array soldered to a custom-made breakout board enabling measurements of the intracochlear electrical field performed in an adjacent study.

### Scanner and imaging parameters

**Figure 1:**
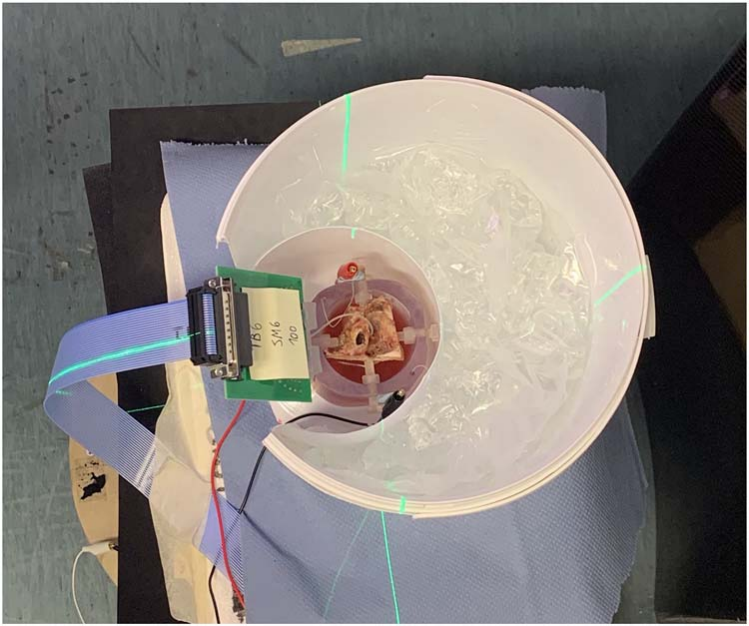
A temporal bone in Ringer’s solution bath enclosed by a water-sac placed in the prototype XFi (Planmeca Oy, Helsinki, Finland) CBCT device.

Figure 1 depicts the temporal bones in the bath placed in the novel full body CBCT device (XFI; Planmeca Oy, Helsinki, Finland). The XFI scanner was used for the imaging of the cadaver temporal bones. The novel rise mechanism of the device offers a unique possibility for both lying and standing full body imaging setting and the latter was used. The tube voltage is adjustable between 80–140 kVp, and the tube load can be adjusted between 10 and 1100 mAs depending on the tube voltage and frame number. The FOV size is adjustable between 50 mm (diameter) × 50 mm (length) and 400 × 200 mm using the offset imaging protocol.

In this study, six different imaging protocols were used for the image acquisition. Furthermore, 50 × 50 mm FOV and 0.3 mm focus size were used to obtain the best image quality. Three of the five scanning protocols were performed using 80, 90 and 100 kVp at constant ED by normalising the ED by adjusting the mAs for each tube voltage (kVp) accordingly. The kVp range was chosen based on a previous study^(10)^, where the best image quality was observed when using 88 kVp. Furthermore, the image acquisitions were also performed for 90 and 100 kVp using 450 mAs to improve the contrast-to-noise ratio (CNR) of the images. The ED and CNR of each scanning protocol were acquired with scanning a phantom with the XFI scanner. The scanning protocols are presented in Table 1.

### Dosimetry

**Figure 2:**
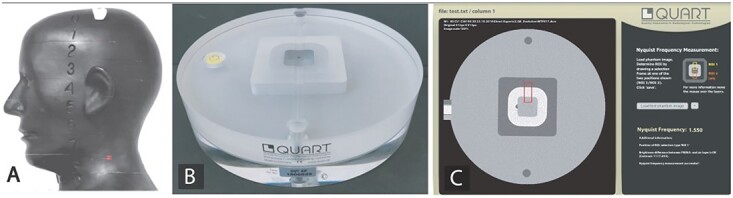
(**A**) Anthropomorphic RANDO RAN 102 phantom used for ED assessments. (**B**) QUART DVT phantom. (**C**) Analysis software window for calculating CNR.

The absorbed organ doses used for ED calculations were acquired using anthropomorphic Rando RAN 102 phantom representing an average man with a length and weight of 175 cm and 73.5 kg, respectively (Radiation Analogue Dosimetry System; Phantom Laboratory, Salem, NY, USA). The phantom comprises a human skull embedded in a soft tissue equivalent material to match the attenuation and scattering conditions of the bone, soft tissues and airways of the human head. The phantom is shown in Figure 2A and consists of 10 25 mm thick layers numbered from 0 to 9 in the order from the calvaria to the neck area. Metal oxide semiconductor field effect transistor (MOSFET) detectors were positioned in the organs as follows: anterior calvarium, midbrain, pituitary fossa, right orbit, right lens, right cheek, right parotid gland, left parotid gland, right mandibular ramus, left mandibular ramus, center cervical spine, left back neck, right mandibular body, left mandibular body, right submandibular gland, left submandibular gland, center sublingual gland, midline thyroid gland, thyroid surface and pharyngeal-esophageal space according to a previous study by Koivisto *et al*.^(13)^.

**Table 1:** The exposure parameters of the imaging protocols.

Protocol nr.	1	2	3	4	5	6^a^
Tube voltage (kVp)	80	90	100	90	100	90
Tube load (mAs)	450	280	200	450	450	450
Tube current (mA)	32	40	40	36	32	36
Exposure time (s)	14.1	7	5	12.5	14.1	12.5
Voxel (μm^3^)	75	75	75	75	75	75
Scan angle (degrees)	210	210	210	210	210	210
Pulsed	Yes	Yes	Yes	Yes	Yes	Yes
Frame number	500	500	500	500	500	500
FOV height (mm)	50	50	50	50	50	50
FOV diameter (mm)	50	50	50	50	50	50

^a^The imaging parameters are the same in the Protocols 4 and 6, but no water-sac was used in the latter.

The phantom was fixed at the same position during all exposures. For all organ dose measurements, a mobile TN-RD-70-W20 MOSFET device was used. In order to calculate the equivalent dose or radiation-weighted dose *H_T_* for all organs or tissues *T*, the following equation was used^(5, 14)^:


$$ {H}_T={w}_R\sum_i{f}_{\mathrm{i}}\times{D}_{Ti} $$

where the radiation weighing factor *w_R_* (Sv/Gy) is equal to 1 for X-rays, *f_i_* is the mass fraction of tissue *T* in layer *i* and *D_Ti_* is the average absorbed dose of tissue *T* in layer *i*. The summation of equivalent dose contributions was done for all phantom layers between 0 and 9.

The ED was obtained from the measured organ doses using the revised guidelines given by the ICRP 103^(15)^. The ED is calculated using the following equation:


$$ ED=\sum_T{w}_T\times{H}_T $$

where *w_T_* is the weighting factor of tissue *T* and *H_T_* is the equivalent dose in tissue *T*.

### Technical image quality and figure-of-merit

The technical image quality indicators CNR and modulation transfer function (MTF) of each protocol were acquired according to Ludlow *et al*.^(16)^ using a QUART DVT_AP phantom (Figure 2B) and QUART DVT_TEC (QUART GmbH, Zorneding, Germany)^(16)^. The phantom consists of 16 cm diameter cylindrical slabs of Plexiglas with PVC and air elements configured to permit measurements of CNR and MTF 10% based on the DIN6868-16 standard^(17)^. Results were calculated from the measurements in a user guided, semi-automatic manner from DICOM slices selected from the volume. Three DICOM slices of each volume were measured, and the results were averaged.

**Table 2:** Computed CNR, MTF and ED for the imaging protocols.

Protocol nr.	1	2	3	4	5	6^a^
CNR	10.5	10.3	10.1	12.4	14.2	12.4
MTF	1.5	1.5	1.6	1.5	1.4	1.5
FOM^b^	2.1	2	1.8	1.8	1.6	1.8
ED (μSv)	52.8	53.8	56	86.5	125.9	86.5

^a^The imaging parameters are the same in the Protocols 4 and 6, but no water-sac was used in the latter.

^b^FOM = CNR^2^/ED.

In this study, the figure-of-merit (FOM) value was calculated to assess the diagnostic efficacy of the image quality versus the ED obtained using different imaging protocols. The FOM value was calculated using the following equation described by Ogden *et al*.^(18)^:


$$ \mathrm{FOM}=\frac{{\mathrm{CNR}}^2}{\mathrm{ED}} $$

### Clinical image quality assessment and statistics

All images acquired with the six different imaging protocols (Table 1) were viewed in the Planmeca Romexis software (Planmeca Oy, Helsinki, Finland). Seven landmarks of the inner ear were evaluated: the cochlea, the modiolus, the osseus spiral lamina, the stapes footplate, the round window niche and the diameter from either the round window or the modiolar wall to the most basal electrode. Three observers, of which two were otosurgeons (with 15 and 8 y of CI surgery experience) and one was a clinical engineer (15 y of experience with CI), graded the image quality from 5 (outstanding image quality) to 1 (the structure is not identifiable) as described by Zou *et al*.^(10)^. Grades 4, 3 and 2 indicated good delineation of the structure, the structure can be evaluated with extra carefulness and the structure can be identified without the details being assessable, respectively. All the observers evaluated the images simultaneously in one viewing session; thus, the circumstances were the same for all. The landmarks were shown to the observers one bone and imaging protocol at a time, and the imaging protocols were presented in a randomised order. The mean and standard deviation (SD) of the observers’ image quality ratings of each structure over the three observers were calculated. The relative interobserver variability was calculated as described by Popović and Thomas^(19)^: $2\times \frac{\mid A-B \mid }{A+B}$, where *A* and *B* are either evaluations of Observers 1 and 2, Observers 1 and 3 or Observers 2 and 3, respectively, and mean of interobserver variability was calculated over the temporal bones. Furthermore, the imaging protocol and subjective image quality was analysed statistically with IBM SPSS 27 (IMB, Armonk, New York) using a Kruskal–Wallis test between imaging protocol and grade. The interobserver variabilities among the three observers were grouped by imaging protocol or evaluated structure and statistically analysed with the Kruskal–Wallis test. The post-hoc comparisons were corrected with the Bonferroni method. A Spearman’s rank correlation coefficient was computed between the mean subjective image quality and technical image quality of each imaging protocol.

## Results

In this study, a technical image quality was computed for six imaging protocols with different imaging parameters. Furthermore, the imaging protocols were used to scan six temporal bones inserted with a CI electrode array, and three independent observers evaluated the quality of the images taken with each protocol. The CNR, MTF, FOM and ED of the different protocols are shown in Table 2. With Protocols 1–3 similar technical image qualities can be acquired with equivalent EDs (CNRs 10.5, 10.3 and 10.1; EDs 52.8, 53.8 and 56.0 μSv, respectively). The technical image quality in the rest of the protocols is superior to Protocols 1–3, but also predispose to higher EDs and lower FOM values.

**Figure 3:**
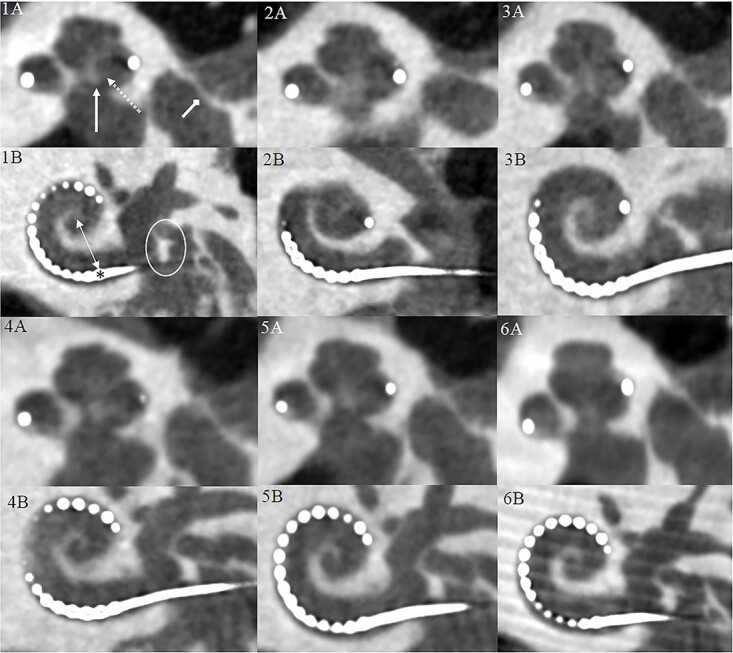
The inner ear structures evaluated from the CBCT images. Two views of each imaging protocol, which is indicated by the number in the upper left corner of each figure, are presented. The osseus spiral lamina (dashed arrow), the modiolus (solid arrow) and stapes footplate (diamond-headed arrow) can be evaluated from the view ‘A’. The most basal electrode (asterisk) whose distance from the round window and the modiolar axis (two-headed arrow) as well as the round window niche (encircled) can be assessed from the view ‘B’. The cochlea and the modiolus (A, solid arrow) can be evaluated from both views.

To assess the utility in clinical practice, the subjective image quality of the imaging protocols was evaluated by three independent observers. Figure 3 demonstrates the seven landmarks of the inner ear evaluated with the different imaging protocols. The mean ± SD of the observers’ image quality ratings of each structure in the different protocols are shown in Table 3 and the relative interobserver variability in Table 4. When the overall subjective image quality of the structures evaluated among the imaging protocols were analysed with the Kruskal–Wallis test, the grades among the imaging protocols were different (*H*(5) = 32.0, *p* < 0.001). A pair-wise comparison of the overall subjective image quality between the protocols shows that the image quality was worse with Protocol 2 when compared with Protocols 5 and 6 (3.0 versus 3.5 and 3.6, respectively, *p* < 0.01 for both). Also, the mean image quality of Protocols 1 (3.2) and 3 (3.1) was worse than the mean image quality of the Protocol 6 (3.6; *p* = 0.021 and *p* = 0.002, respectively). Furthermore, a similar analysis between the interobserver variability among the imaging protocols was conducted with the Kruskal–Wallis test. No differences in interobserver variability between the imaging protocols were detected (*H*(5) = 6.81, *p* = 0.235). The mean ± SD interobserver variability of each imaging protocol was 0.29 ± 0.25, 0.32 ± 0.27, 0.25 ± 0.26, 0.28 ± 0.26, 0.25 ± 0.21 and 0.29 ± 0.28 for Protocols 1–6, respectively. Finally, the interobserver variability between the evaluated structures was analysed with the Kruskal–Wallis test and a significant difference was detected (*H*(6) = 50.8, *p* < 0.001). The mean ± SD interobserver variability was 0.22 ± 0.19 for cochlea, 0.26 ± 0.22 for modiolus, 0.41 ± 0.37 for osseus spiral lamina, 0.23 ± 0.20 for round window niche, 0.19 ± 0.18 for most basal electrode, 0.33 ± 0.27 for modiolar distance and 0.28 ± 0.26 for stapes footplate. The pair-wise comparisons after the Bonferroni correction showed significant differences in interobserver variability for cochlea versus osseus spiral lamina and stapes footplate (*p* < 0.001 and *p* = 0.03), osseus spiral lamina and round window niche (*p* = 0.008), and most basal electrode versus modiolus, osseus spiral lamina, basal modiolar distance and stapes footplate (*p* < 0.05 for all comparisons). These results indicate that the greatest disagreement of the visualisation of a structure between the observers was for structures that were the hardest to visualise.

**Table 3:** Clinical image quality demonstrated by average levels of three observers using a rating system from 5 to 1 in descending order. The mean ± SD of the observers’ image quality ratings of each structure over the observers are presented in each cell.

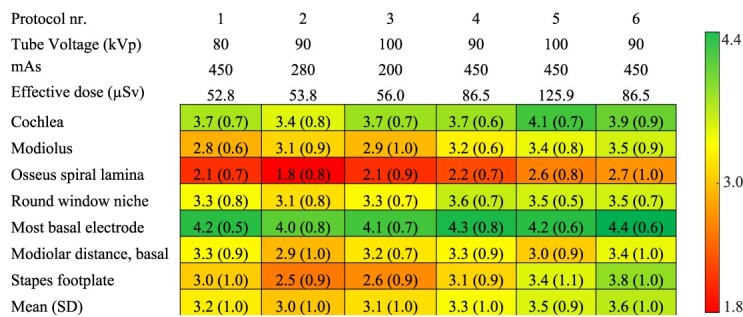

**Table 4:** The relative interobserver variability. The first, second and third number in each cell is the mean absolute relative difference in the evaluations over the temporal bones between the otosurgeons, between the Otosurgeon 1 and clinical engineer, and between the Otosurgeon 2 and the clinical engineer, respectively.

Protocol nr.	1	2	3	4	5	6
Cochlea	0.23, 0.18, 0.22	0.35, 0.27, 0.08	0.13, 0.19, 0.23	0.24, 0.28, 0.13	0.22, 0.25, 0.11	0.20, 0.44, 0.24
Modiolus	0.27, 0.30, 0.16	0.37, 0.36, 0.15	0.29, 0.1, 0.26	0.27, 0.32, 0.14	0.27, 0.32, 0.13	0.34, 0.46, 0.12
Osseus spiral lamina	0.52, 0.56, 0.31	0.62, 0.4, 0.46	0.58, 0, 0.58	0.46, 0.63, 0.2	0.28, 0.27, 0.28	0.38, 0.64, 0.27
Round window niche	0.29, 0.32, 0.25	0.34, 0.25, 0.27	0.23, 0.23, 0.1	0.26, 0.34, 0.18	0.19, 0.19, 0.19	0.16, 0.25, 0.18
Most basal electrode	0.23, 0.05, 0.19	0.30, 0.18, 0.20	0.28, 0.05, 0.23	0.30, 0.26, 0.11	0.24, 0.13, 0.19	0.19, 0.16, 0.11
Modiolar distance, basal	0.26, 0.48, 0.29	0.42, 0.38, 0.28	0.10, 0.19, 0.19	0.39, 0.4, 0.18	0.39, 0.43, 0.20	0.55, 0.58, 0.04
Stapes foot plate	0.42, 0.21, 0.43	0.40, 0.16, 0.47	0.46, 0.31, 0.49	0.33, 0.11, 0.31	0.48, 0.26, 0.22	0.39, 0.24, 0.22

Furthermore, the image quality of single structures was compared among protocols (Table 3). When the image quality of osseus spiral lamina was grouped by imaging protocol, the Kruskal–Wallis test revealed differences among the imaging protocols (*H*(5) = 11.2, *p* = 0.048). In pair-wise comparison without the Bonferroni correction, the image quality was worse in the Protocol 2 when compared with Protocols 5 and 6 (*p* = 0.11 and *p* = 0.005). However, when Bonferroni correction was applied, the image quality of the osseus spiral lamina was similar between the groups. Also, the Kruskal–Wallis test detected differences in the image quality of the stapes footplate between the imaging protocols. The mean image quality of the stapes footplate was worse in Protocols 2 and 3 when compared with Protocol 6 (*p* = 0.006 and *p* = 0.014, respectively). When similar analyses were conducted for the image quality of the cochlea, the modiolus, the round window niche, the most basal electrode and the basal modiolar distance, no significant differences were found between the protocols. These results demonstrate that most of the inner ear structures can be visualised with low-dose imaging protocols. However, when the most delicate structures, such as the osseus spiral lamina or stapes footplate need visualisation, a high dose imaging protocol might be required. Overall, protocol number one provided similar image quality as the clinically relevant high-dose protocols.

**Figure 4:**
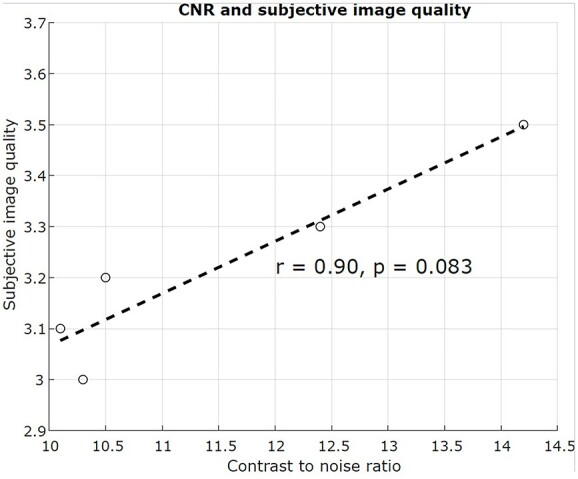
Subjective image quality (*y*-axis) plotted against CNR (*x*-axis). The dotted line depicts linear regression between the subjective image quality and CNR with corresponding Spearman’s rank correlation coefficient (*r* = 0.90, *p* = 0.083). Protocol 6 was excluded from the analysis, as it is not clinically relevant because of the lack of the water-sac.

Figure 4 demonstrates the relationship between the subjective image quality and the CNR of Protocols 1–5, as the clinically irrelevant Protocol 6 was excluded from the analysis. A strong Spearman’s rank correlation was found between the two, however, without statistical significance (*r* = 0.90, *p* = 0.083). The result demonstrate that the subjective image quality could be predicted from the technical image quality.

**Figure 5:**
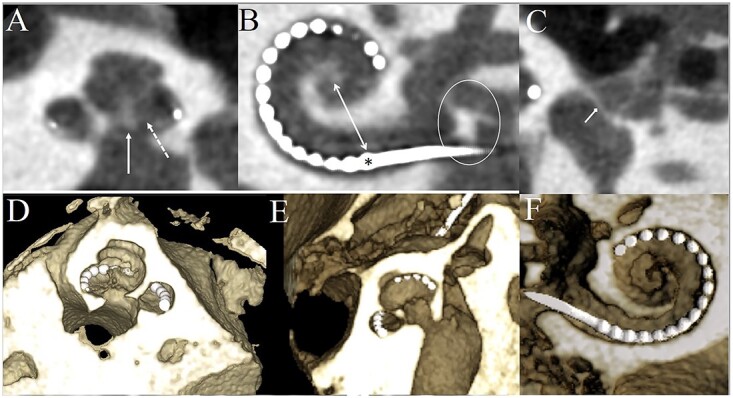
Inner ear structures imaged with the optimised low-dose imaging protocol. The upper panel (**A**–**C**) shows the anatomy in 2D planes. The osseus spiral lamina is marked with a dashed arrow, the modiolus with a solid arrow, the two-headed arrow points distance between the electrode and the modiolus, the round window niche is encircled with a white circle and the stapes footplate is marked with a diamond-headed arrow. The lower panel (**D**–**F**) shows the cochlea inserted with a CI electrode array in 3D reconstructions.

Figure 5 shows inner ear structures imaged with Protocol 1. From the upper panel (Figure 5A–C), the cochlea, the osseus spiral lamina, the round window niche, the modiolar distance and the location of the electrode array can be evaluated from 2D planes. The lower panel (Figure 5D–F) depicts the complex anatomy of the cochlea in 3D reconstructions.

## Discussion

The purpose of this study was to optimise the imaging parameters for a novel CBCT device in inner ear imaging when a CI electrode array is placed in the cochlea. In line with Zou *et al*.^(10)^, who investigated the usefulness of an experimental CBCT set-up version of the XFi device in inner ears with a CI electrode array, the results of this study indicate, that the XFi scanner is useful in intraoperative or postoperative imaging of CI recipients.

As this is a cadaver temporal bone study, to emulate a real clinical setting, the bones were enclosed in a water-sac simulating the brain and the soft tissues of the head. The imaging Protocols 5 and 6 had the best technical and subjective image qualities but also caused the highest radiation exposures to the specimen. However, the performance of Protocol 6 has no clinical relevance, as no water-sac was used. Overall, when taking both subjective image quality and ED into account, Protocol 1 (80 kVp, 450 mAs) is preferred as it provides better, even though not statistically significant, image quality of the smallest structures than the other low ED protocols and smaller EDs than the high ED protocols. The ED of the imaging Protocol 1 is only 52.8 μSv, which is equivalent to approximately three chest X-rays or three dental panoramic radiographs^(20)^.

The osseus spiral lamina is a sheer bony structure dividing the cochlear duct into ST and SV, whose visualisation could be helpful when assessing possible scalar translocation of the electrode array. Unfortunately, the osseus spiral lamina is arguably the most challenging structure to visualise in the inner ear^(21–23)^. In our study, the osseus spiral lamina was depicted with moderate results with both the optimised low-dose and high-dose imaging protocols. Also, the delineation of the stapes footplate is generally poor with MDCT^(23)^. However, in our study, the stapes footplate can be evaluated from CBCT images with the optimised protocol in similar fashion as from CBCT images imaged with the high-dose protocols.

Even though the three examiners were experienced clinicians and did their evaluations independently, the number of evaluations of each protocol was quite small, only six specimens per evaluated structure. Also, the clinicians work in the same clinic and might be used to the same advantages and disadvantages of the CT device currently in clinical use. Therefore, more examiners with different backgrounds could provide different results.

In conclusion, with the novel CBCT device, even the most delicate structures of the CI recipient’s inner ear, such as the stapes footplate and the osseus spiral lamina, can be visualised. When the imaging parameters are selected carefully, the most relevant inner ear structures can be depicted with a small ED. However, if there is a need to scrutinise the stapes footplate or osseus spiral lamina, an imaging protocol with better image quality and higher ED than with the low ED protocol is likely required.

## Funding

This research did not receive any specific grant from funding agencies in the public, commercial or not-for-profit sectors.

## Declaration of competing interest

None.
